# Graphene Aerogel Composites with Self-Organized Nanowires-Packed Honeycomb Structure for Highly Efficient Electromagnetic Wave Absorption

**DOI:** 10.1007/s40820-024-01541-y

**Published:** 2024-10-21

**Authors:** Xiao You, Huiying Ouyang, Ruixiang Deng, Qiuqi Zhang, Zhenzhong Xing, Xiaowu Chen, Qingliang Shan, Jinshan Yang, Shaoming Dong

**Affiliations:** 1 State Key Laboratory of High Performance Ceramics and Superfine Microstructure, Shanghai Institute of Ceramics, Chinese Academy of Sciences, Shanghai, 200050 People’s Republic of China; 2 Key Laboratory of Inorganic Coating Materials CAS, Shanghai Institute of Ceramics, Chinese Academy of Sciences, Shanghai, 200050 People’s Republic of China; 3 Structural Ceramics and Composites Engineering Research Center, Shanghai Institute of Ceramics, Chinese Academy of Sciences, Shanghai, 200050 People’s Republic of China; 4 Center of Materials Science and Optoelectronics Engineering, University of Chinese Academy of Sciences, Beijing, 100049 People’s Republic of China; 5 School of Physical Science and Technology, ShanghaiTech University, Shanghai, 201210 People’s Republic of China; 6 School of Materials Science and Engineering, Zhejiang Sci-Tech University, Hangzhou, 310018 People’s Republic of China; 7 University of Chinese Academy of Sciences, Beijing, 100039 People’s Republic of China

**Keywords:** Hierarchical porous structure, Interface, High-temperature resistance, Graphene aerogel composites, Electromagnetic wave absorption

## Abstract

With vigorous developments in nanotechnology, the elaborate regulation of microstructure shows attractive potential in the design of electromagnetic wave absorbers. Herein, a hierarchical porous structure and composite heterogeneous interface are constructed successfully to optimize the electromagnetic loss capacity. The macro–micro-synergistic graphene aerogel formed by the ice template‑assisted 3D printing strategy is cut by silicon carbide nanowires (SiC_nws_) grown in situ, while boron nitride (BN) interfacial structure is introduced on graphene nanoplates. The unique composite structure forces multiple scattering of incident EMWs, ensuring the combined effects of interfacial polarization, conduction networks, and magnetic-dielectric synergy. Therefore, the as-prepared composites present a minimum reflection loss value of − 37.8 dB and a wide effective absorption bandwidth (EAB) of 9.2 GHz (from 8.8 to 18.0 GHz) at 2.5 mm. Besides, relying on the intrinsic high-temperature resistance of SiC_nws_ and BN, the EAB also remains above 5.0 GHz after annealing in air environment at 600 °C for 10 h.

**Supplementary Information:**

The online version contains supplementary material available at 10.1007/s40820-024-01541-y.

## Highlights

- A new strategy for elaborate regulation of microstructure was successfully introduced by the ice template‑assisted 3D printing and chemical vapor deposition strategy, including graphene nanoplate/silicon carbide nanowires hierarchical porous structure and graphene nanoplate/boron nitride composite heterogeneous interface.
- The composite exhibits excellent electromagnetic wave absorption performance with an *RL*_min_ of -37.8 dB and an EAB_max_ of 9.2 GHz (from 8.8 to 18.0 GHz) at 2.5 mm. And the high-temperature absorption stability makes it a promising absorber candidate under high temperature and oxidizing atmosphere.

**Supplementary Information:**

The online version contains supplementary material available at 10.1007/s40820-024-01541-y.

## Graphical abstract

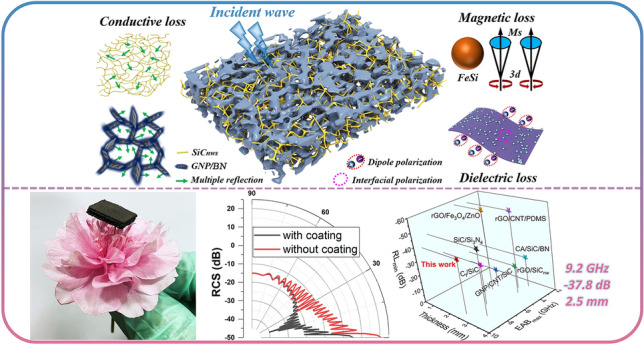

## Introduction

With the remarkable improvement of the cooperative strike capability of space detection and strategic defense systems in recent years, the multi-temperature and wide-band wave absorption technology of aerospace and weapon equipment shows great application potential in the field of “radar stealth” technology [1–3]. Inspired by honeycomb structure and spider structure in nature, the construction of porous structures is the main direction in structural regulation of electromagnetic absorbing material [4]. The porous structure can improve the impedance matching degree, and the connected pores formed are conducive to the entry of incident electromagnetic waves (EMWs) into the absorbers [5–7]. Meanwhile, the multiple scattering of EMWs in the pore structure can also significantly increase the loss capacity in the transmission process [8–10]. On this basis, more innovative hierarchical porous structures are also applied to optimize absorption performance [11, 12]. The introduction of micro-scale pores intensifies the scattering intensity of incident EMWs, while the high specific surface area of nanoscale pores enhances the dissipation of EMWs by improving the polarization relaxation ability [13, 14]. Therefore, it is predictable that the elaborate design of the interior morphology of absorbers will continue to be practiced toward advanced performance, and further studies related to hierarchical porous structures deserve more attention.

Low-dimensional nanomaterials are the reasonable choice for constructing hierarchical porous structures [15, 16]. Considering the high specific surface area, abundant stacking faults, and twinning interfaces, the adjustable dielectric properties are the key points to realize the coupling optimization of impedance matching degree and loss capacity [17, 18]. For one-dimensional materials, such as silicon carbide nanowires (SiC_nws_) and carbon nanotubes (CNTs) with high aspect ratio, the carrier path excited by the electric field can be larger [19–22]. Moreover, the overlapped through network structure further enlarges the transmission path of incident EMWs and enhances the conductive loss [23, 24]. For two-dimensional materials with a high aspect ratio, the most representative graphene nanoplates (GNPs) and MXene materials are easy to form connected pore structures, so that the incident EMWs can be scattered and reflected inside the micropore and between the nanoplates [25–29]. Wang et. al fabricated an ultralight SiC_nw_@SiC foam with highly efficient microwave absorption, which could be attributed to the interface polarization and dielectric loss by the construction of SiC skeleton [30]. Yin et. al prepared absorbing composites with effective absorption in the entire X-band by means of incorporating SiC nanowires into rGO foams, which revealed the dielectric loss of SiC_nws_ and conductive loss of interconnected graphene networks [31]. The hierarchical porous structure mentioned above inspires us to construct multi-dimensional materials to increase scattering efficiency and introduce composite electromagnetic loss mechanisms. More importantly, considering the potential harsh application environments of structural absorption composites, the binding states and distribution rules of multi-phase are also worth further exploration.

Herein, a new strategy for elaborate regulating of microstructure was introduced successfully by the ice template‑assisted 3D printing and chemical vapor deposition (CVD) strategy, including GNP/SiC_nws_ hierarchical porous structure and GNP/boron nitride (BN) composite heterogeneous interface. The porous “cage structure” can induce multiple scattering of incident EMWs, and the composite loss mechanism originating from the formed interfacial structure ensures excellent absorption performance with a wide effective absorption bandwidth (EAB) of 9.2 GHz. On this basis, the BN interface effectively delays the oxidation rate of GNP and endows as-prepared composites with structure and wave absorption stability at high temperatures. This work may serve as one of the design guidelines for the collaborative optimization design of the structure and function of low-dimension materials.

## Experimental Section

### Preparation of Printable Inks

Graphene oxide (GO, 10–50 μm, Tanfeng Tech. Inc), ferroferric oxide (Fe_3_O_4_, 10–30 nm, XFNANO Tech. Inc), and nickel nitrate (Ni(NO_3_)_2_·6H_2_O, Sinopharm Chemical Reagent CO. Ltd.) were mixed in deionized water, followed the sonication process (200 W, Scientz-IID, Ningbo Scientz Biotechnology CO. Ltd.) was applied to realize uniform dispersion of each component. The self-supporting printable inks were prepared by removing excess solvent in a water bath. Their viscosity and rheological properties could be monitored by adjusting the amount of residual solvent. Additionally, pasty graphene oxide inks with a graphene oxide concentration of 200 mg mL^−1^ were prepared.

### RCS Simulation

A commercial high-frequency simulator was used for simulation. In the simulation process, the bottom layer of the model is a metal plate, set as the material of PEC (perfect electric conductor), and the size is 300 mm × 300 mm × 1 mm. Above the metal plate is a microwave absorber, the size of which is 300 mm × 300 mm × 3 mm. The complex dielectric constant and permeability frequency measured by sample GBS_2_ are used as the electromagnetic parameters of the microwave absorber. The variation of the radar cross section (RCS) of the metal plate with the incidence angle and frequency before and after the microwave absorber is simulated. The incidence angle varies from  − 90° to 90°, and the step is 1°. The frequency range is 2–18 GHz, and the step frequency is 0.1 GHz.

### Construction of 3D Customized GO Aerogel

The high-modulus GO inks were loaded into a syringe barrel that was mounted onto a three-axis positioning stage (3D bioprinter V2.0, Regenovo Biotechnology Co., China), and customized GO lattice was reproduced according the input 3D model with a size of 15 mm × 15 mm × 2.5 mm. The GO lattice was composed of 5 densely stacked printed layers and the thickness of the adjacent layers are set to 0.5 mm. After printing, the 3D GO lattice was immersed in liquid nitrogen (− 196 °C) for 10 min to implement frozen self-assembly of GO nanoplates and then transferred into a freezer (− 60 °C) for 48 h to further ice crystallization. Subsequently, 3D GO aerogel was obtained with a drying process (− 60 °C) for 48 h to construct a macro-regular and micro-porous network structure.

### Synthesis of 3D Graphene/BN/SiC_nws_ Composites

BN and SSiC_nws_ were introduced by a typical CVD process. The self-supporting GA was prepared by thermal annealing of GO aerogel at 800 °C for 1 h under the argon atmosphere, and BN was deposited on graphene nanoplates by the reaction of boron trichloride (BCl_3_) and ammonia (NH_3_). Then, the temperature rose to 1100 °C and continuous SiC_nws_ was introduced by cracking methyltrichlorosilane (MTS, CH_3_SiCl_3_) with the presence of Ni ions. In this work, the deposition time of BN interface was set to 1 h, and the thickness was ~ 100 nm. By adjusting the volume fraction of SiC_nws_, including 2 and 4 h deposition time, the as-prepared samples were labeled GS_2_, GBS_2_, and GBS_4_ composites.

### Characterization

The morphology, structure, and composition of as-prepared composites were characterized by a field-emission scanning electron microscopy (FESEM, Hitachi SU8220, Japan), a transmission electron microscopy (TEM, JEM-2100F, Japan), an X-ray diffractometer (XRD, Rigaku Corporation, Kyoto, Japan), a laser micro-Raman spectroscopy (Thermo Nicolet, USA), and an X-ray photoelectron spectroscopy (XPS, Thermofisher scientific 250Xi). The electromagnetic parameters (*ε*_*r*_ and *μ*_*r*_) in 2–18 GHz of as-prepared composites were measured by coaxial transmission line method on a vector network analyzer (VNA, ZVB 20, Rohde & Schwarz, Germany). The test samples were laser cut into an annulus with an inner diameter of 3 mm, an external diameter of 7 mm, and a thickness of 2 mm. All the measurements on the vector network analyzer were calibrated by the standard SOMT method.

## Results and Discussion

### Design Principle and Preparation of 3D Graphene/BN/SiC_nws_ Composites

The special bio-structures in nature generally provide creative ideas for the macro-assembly of low-dimensional nanomaterials, such as the lamellar aerogel structure inspired by the honeycomb structure and the emitting nanowire structure inspired by the dandelion structure. The biomimetic structures are endowed with novel functions, including electromagnetic absorption, thermal insulation, and noise reduction. For wave absorption applications, both the lamellar honeycomb structure and the emitting nanowire structure mentioned above can endow multiple scattering and loss of incident electromagnetic waves. Innovatively, the mechanism inspired us to pack the pores of the lamellar honeycomb structure with an emitting nanowire structure, which will inevitably increase the scattering capacity for microwaves, thereby optimizing the wave absorption performance.

**Fig. 1:**
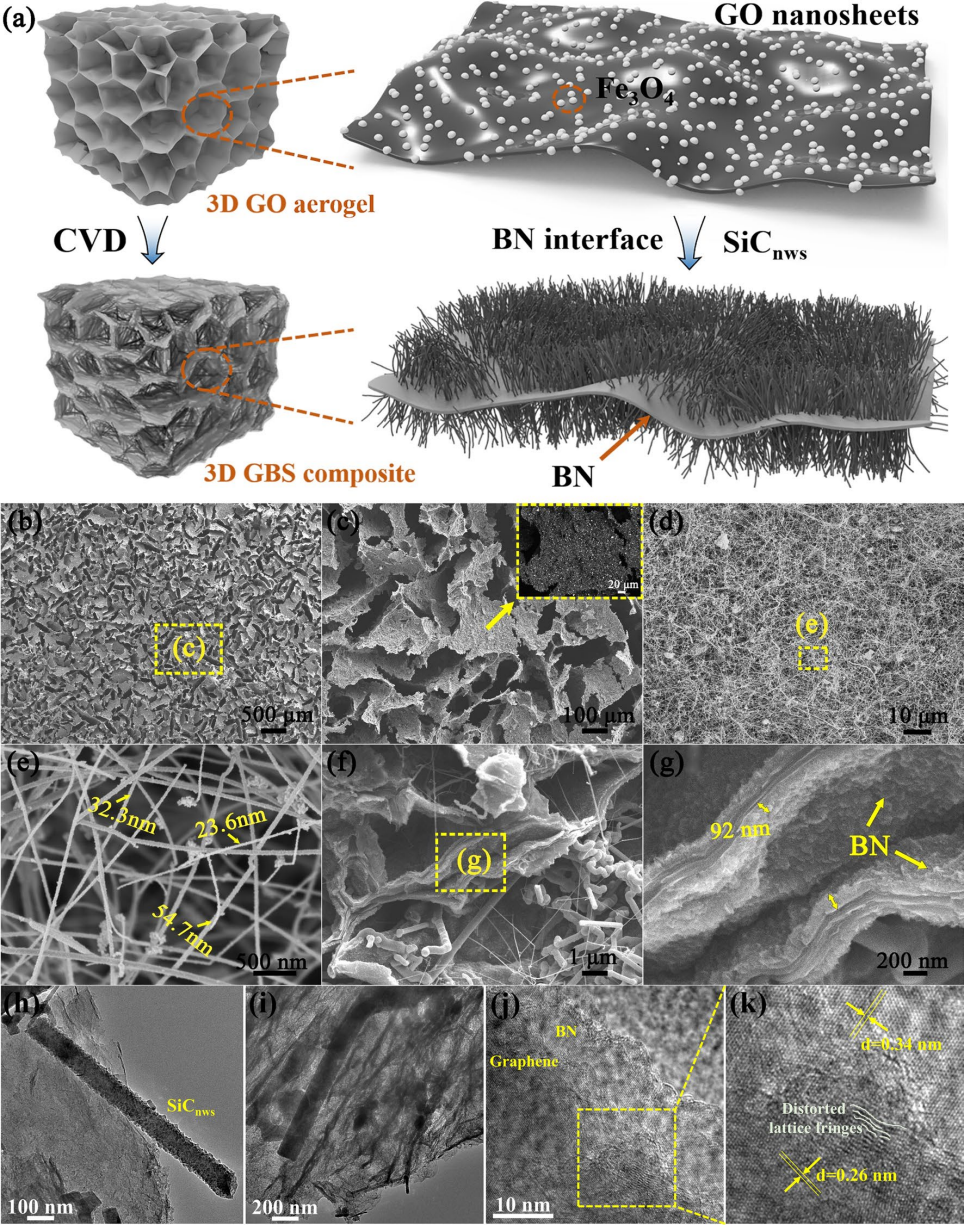
**a** Schematic illustration of as-prepared GBS composites. **b**, **c** Microstructure of as-prepared GA composites and the inset is a backscattered view that shows the uniform distribution of Fe_3_O_4_. FESEM images of **d**, **e** the exterior surface view and **f**, **g** the fracture surface view of as-prepared GBS_2_ composites. **h**, **i** TEM images and **j**, **k** HRTEM images of as-prepared GBS_2_ composites

In this work, in view of the easy oxidization of graphene nanoplates at high temperatures, the fine-tuning of the microstructure has been applied to optimize the high-temperature absorbing performance of composites. To assemble GO nanoplates into a regular 3D porous architecture, a creative construction routine is developed by integrating 3D printing ice and freeze casting. Due to the hydrophilicity and strong gelation capability of GO nanoplates, stable aqueous printable inks with high viscosity and shear thinning behavior can be easily obtained. Considering the structural designability of 3D printing, the macro-configuration of the 3D GO lattice can be monitored by on-demand stacking. The process guarantees the consistent and reliable formation of the GO skeleton, thereby enabling the subsequent construction of hierarchical porous structures that are conducive to the absorption of electromagnetic waves. Subsequently, the freeze casting process is applied to build an interconnected GO network with a porous structure, which originates from the self-assembly of GO nanoplates induced by frozen ice crystals. Meanwhile, Fe_3_O_4_ nanoparticles are uniformly adsorbed on the GO nanoplates due to the huge specific surface area, as shown in Fig. 1a. After followed thermal annealing at 800 °C to prepare reduced GO aerogel, while BN interface and SiC_nws_ are introduced in situ by CVD method. The wave-transmitting phase BN will significantly increase the transmission path of EMWs, thereby increasing the loss. Due to the low dielectric constant of BN, the combination with graphene can realize complementary dielectric properties and optimize the impedance matching degree. Notably, the high-temperature resistance of BN and SiC_nws_ can effectively inhibit the oxidation behavior of graphene in a high-temperature environment, and endow the composites with stable high-temperature absorption performance.

### Structure, Microscopic Morphology, and Composition

The microstructure of 3D GO aerogel and 3D GBS composites are illustrated in Fig. 1b–g. The freeze casting process in liquid nitrogen facilitates the growth of ice crystal, and the GO nanoplates at the edge of the ice crystal is squeezed to realize self-assembly. Due to the π–π interactions, the nanoplates are interconnected in a honeycomb porous structure. The size of the pores is approximately a few micrometers (≈ 48.9 μm). Meanwhile, the inset in Fig. 1b shows the regular 3D lattice structure constructed by 3D printing. The backscattered view of GO nanoplate in Fig. 1c presents the uniform distribution of Fe_3_O_4_ nanoparticles, which can be attributed to the electrostatic adsorption and electrostatic coordination of positive and negative charges. With the in situ introduction of BN interface, the GO nanoplates are reduced and fully encapsulated. Considering the oxidation resistance of BN over 800 °C, the oxidation behavior of graphene nanoplates at high temperatures can be effectively suppressed. Figure 1d–g shows the fracture surface of 3D GBS composites after the growth of SiC_nws_. It can be obviously seen that lots of nanowires with high aspect ratios are introduced in a random distribution, which about 36.9 nm in diameter (average of 3 different nanowires) and several hundred micrometers in length. The smooth surface structure also demonstrates the high crystallinity of the nanowires. The EDS element mapping of an individual nanowire shows the presence of Si, C, and a small amount of O elements, indicating the main phase of the nanowires is SiC (Fig. S1). As shown in Fig. 1h–k, the SiC_nws_ are found to be grown in situ on the graphene and BN layers, which is in accordance with the SEM results presented in Fig. 1f, g. The HRTEM results indicate that the graphene and BN are well crystallized. The lattice spacing of the graphene and BN is 0.26 and 0.34 nm, respectively, which corresponds to the (002) plane of graphene and the (002) plane of BN. Furthermore, a substantial number of discontinuous and distorted lattice stripes are observed, indicating that the in situ growth of BN introduced a multitude of defects and dipoles, which subsequently augmented the polarization relaxation loss process of the electromagnetic waves [32].

The chemical composition of the GO aerogel, GS composites and GBS composites is characterized. In Fig. S2a, the diffraction peaks at 10.9° and 26.5° are assigned to GO aerogel and reduced GO nanoplates, and the conversion of (001) plane of GO to (002) plane of GNP represents a decrease in the interplanar spacing. The major diffraction peaks of GBS_2_ and GBS_4_ composites are assigned to the (111), (200), (220), (311), and (222) planes of 3C-SiC, and the sharp peaks reveal high crystallinity of SiC nanowires. Notably, the BN characteristic peak cannot be found in all samples. Figure S2b illustrates typical Raman spectra of the composites. Three peaks at around 1343, 1593, and 2660 cm^−1^ can be assigned to the D-band, G-band, and 2D-band of graphene, and the *I*_D_/*I*_G_ value shifts from 0.70 of GO to 2.3 of reduced GO. The increase in *I*_D_/*I*_G_ value corresponds to the introduction of defects in nanoplates during the deposition process, which is beneficial to introduce the loss mechanism of defect polarization. Moreover, the two characteristic peaks located at 775 and 950 cm^−1^ represent the absorption bands from transversal optic and longitudinal optic mode 3C-SiC [33].

The chemical composition and the group distribution are further characterized by XPS. The survey spectrum in Fig. S2c, d shows the presence of Fe, O, C, and Si elements of the GS_2_ composite, and the N element can be found in the GBS_2_ composite. Therefore, the N element proves the effective introduction of BN interphase. However, BN interphase cannot be detected in XRD pattern, which can be attributed to the in situ growth of amorphous BN by the CVD process.

### Microwave Absorption Performance and Mechanism at Room Temperature

The regulation of electromagnetic parameters of the prepared composites by BN interface and SiC_nws_ can directly affect the microwave absorption properties. Therefore, the relative complex permittivity and permeability (*ε*_*r*_ and *μ*_*r*_) and corresponding tangent loss (tan *δ*_*ε*_ and tan *δ*_*μ*_) of 3D GS composites and 3D GBS composites are measured by coaxial transmission line method. According to the Debye theory [34]:

1 $$ \varepsilon_{r} = \varepsilon^{\prime} - j\varepsilon^{\prime\prime} = \varepsilon_{\infty } + \frac{{\varepsilon_{s } - \varepsilon_{\infty } }}{1 + j2f\tau } $$

2 $$ \varepsilon^{\prime} = \varepsilon_{\infty } + \frac{{\varepsilon_{s } - \varepsilon_{\infty } }}{{1 + \left( {2f} \right)^{2} \tau^{2} }} $$

3 $$ \varepsilon^{\prime\prime} = \varepsilon_{p}^{\prime \prime } + \varepsilon_{c}^{\prime \prime } = \frac{{2f\tau \left( {\varepsilon_{s } - \varepsilon_{\infty } } \right) }}{{1 + \left( {2f} \right)^{2} \tau^{2} }} + \frac{\sigma }{{2\pi f\varepsilon_{0} }} $$

in which *ε*_*∞*_, *ε*_s_, *f*, *τ* and $\sigma$ represent the relative dielectric constant at infinite frequency, static dielectric constant, frequency of microwaves, polarization relaxation time and electrical conductivity, respectively. The ε″ represents the ability of the composites to loss electromagnetic energy, which is affected by dipolar relaxation process.

The real and the imaginary parts of the electromagnetic parameters indicate the polarization ability and electromagnetic wave loss ability under electromagnetic field conditions, respectively. These parameters are related to several factors, including the structure and the composition of the composites. The tan *δ*_*ε*_ and tan *δ*_*μ*_ denote the dielectric loss and magnetic loss of the absorber, respectively.

**Fig. 2:**
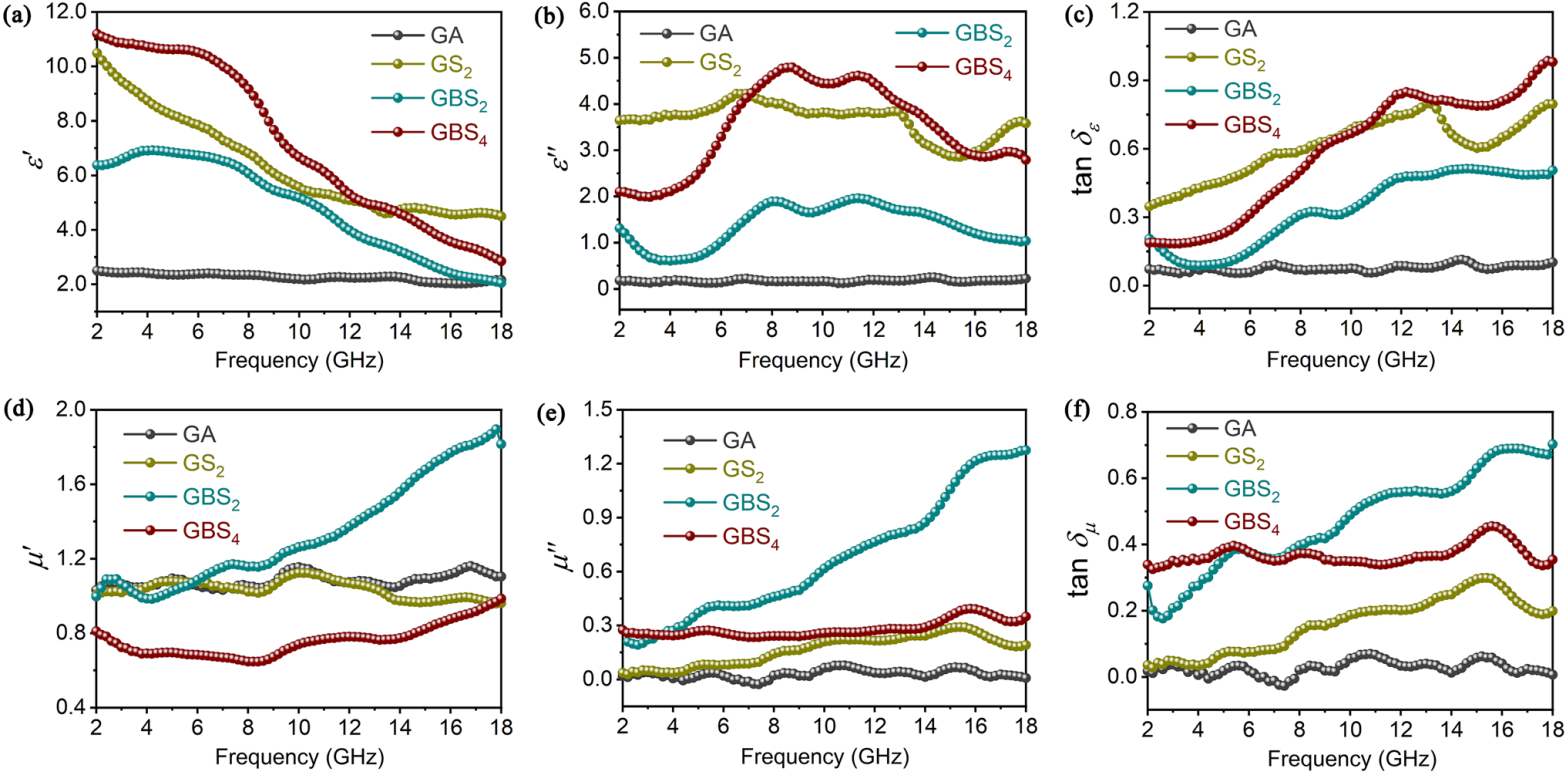
**a** Real part *ε*′, **b** imaginary part *ε*″, and **c** tangent loss tan *δ*_*ε*_ as a function of frequency of as-prepared samples. **d** Real part *μ*′, **e** imaginary part *μ*″, and **f** tangent loss tan *δ*_*μ*_ as a function of the frequency of as-prepared samples

As shown in Fig. 2a, b, the dielectric constant of GO aerogel is comparable to that of air, which is attributed to its highly porous structure. The dielectric constant of each composite increases with the in situ growth of BN and SiC_nws_. The porosity of the GA, GBS_2_, and GBS_4_ composites is measured in Fig. S3. The GA composite exhibits a porosity of 89.9%, with most of the pore sizes distributed at 48.9 µm. This pore size is attributed to the honeycomb structure constructed by the 3D printing and freeze casting process, which is conducive to optimizing the degree of impedance matching. Meanwhile, the expansion of in situ-grown SiC_nws_ has led to a decrease in both the porosity (67.7% and 63.1%) and the pore size of the GBS_2_ and GBS_4_. This further results in a slight increase in the dielectric constant. However, the large number of SiC_nws_ provides an extremely high specific surface area, which enhances polarization loss and conductivity loss. Moreover, the dielectric constants of GBS_2_ were found to be lower than those of GS_2_, indicating that the introduction of BN effectively reduces the migration path of free electrons and prevents the formation of excessive continuous microwave reflection networks. This can reduce the dielectric constant of the composites and optimize the impedance matching performance of the composites.

The dielectric loss process of the composite includes conduction loss and polarization loss [35, 36]. As shown in Fig. S4, the dielectric loss processes of composites are all dominated by the conduction loss, which accounts for more than 90% of the total. This is attributed to the formation of a conductive network by graphene nanoplates interconnections and the higher conductivity of the composites. The fluctuations in the *ε*″ curves are related to the multiple polarization relaxation process induced by interfacial polarization. The fluctuations in *ε*″ curves become more pronounced with increasing time of in situ growth of BN and SiC_nws_. This indicates that in addition to the conductive loss associated with the carbon skeleton, the polarization loss also plays a significant role in microwave absorption. Furthermore, the tan *δ*_*ε*_ of each composite in Fig. 2c is higher than that of GA, which indicates that the introduction of the interfaces and SiC_nws_ can enhance the interfacial polarization and dipole polarization behaviors, which in turn effectively improves the dielectric loss capability of the composites.

As illustrated in Fig. 2d, e, the relative complex permeability of GA and GS_2_ is nearly equal to 1-0j, which suggests a weak magnetic loss. However, the uniform distribution of Fe_3_O_4_ on graphene sheets resulted in the highest magnetism of the GA, whereas the magnetic properties of the composites were significantly reduced following the in situ growth of SiC_nws_ (Fig. S5). This is due to the fact that during the CVI process, Fe_3_O_4_ is reduced to Fe monomers by H_2_, while free Si atoms from MTS cleavage react with Fe to produce the weakly ferromagnetic FeSi phase (consistent with the results in Fig. S2a). Consequently, the magnetism of the composites is weakened with the introduction of SiC_nws_. Furthermore, the magnetism of GBS_2_ and GBS_4_ with in situ-grown BN are superior to those of GS_2_. This is due to the fact that the introduction of BN encapsulates the Fe_3_O_4_ particles, thereby reducing their transition to the FeSi process [37]. Additionally, BN produces an induced magnetic moment in a magnetic field and exhibits weaker para magnetism [38]. Therefore, the composites display soft magnetic characteristics, which align with the results of higher magnetic permeability for GBS_2_ and GBS_4_. Since BN improves the magnetization of the composite, the tan *δ*_*μ*_ of the composites increases with the introduction of BN (as shown in Fig. 2f), and the magnetic loss is enhanced.

Furthermore, the tan *δ*_*ε*_ and tan *δ*_*μ*_ of the GBS_2_ and GBS_4_ composites are more closely aligned than those of the GS_2_ samples, where dielectric loss is the dominant phenomenon (Fig. S6). This suggests that the introduction of BN can effectively regulate the balance between dielectric loss capability and magnetic loss capability, ultimately leading to optimized impedance matching.

The microwave absorption performance of absorbers can be visually expressed by the reflection loss (*RL*) value and the EAB. According to the electromagnetic parameters, the *RL* value is described as follows [39, 40]:

4 $$ RL \left( {dB} \right) = 20 \lg \left| {\frac{{Z_{in} - 1}}{{Z_{in} + 1}}} \right| $$

5 $$ Z_{in} = \sqrt {\frac{{\mu_{r} }}{{\varepsilon_{r} }}} \tanh \left[ {j\frac{2fd}{c}\sqrt {\mu_{r} \varepsilon_{r} } } \right] $$

where *Z*_*in*_ represents the normalized input impedance of the absorbers, *d* and *c* refer to the thickness of the absorbers and the velocity of the microwave in free space. When the *RL* value is less than  − 10 dB, it means that more than 90% incident EMWs can be absorbed by the absorbers, and only less than 10% is reflected.

**Fig. 3:**
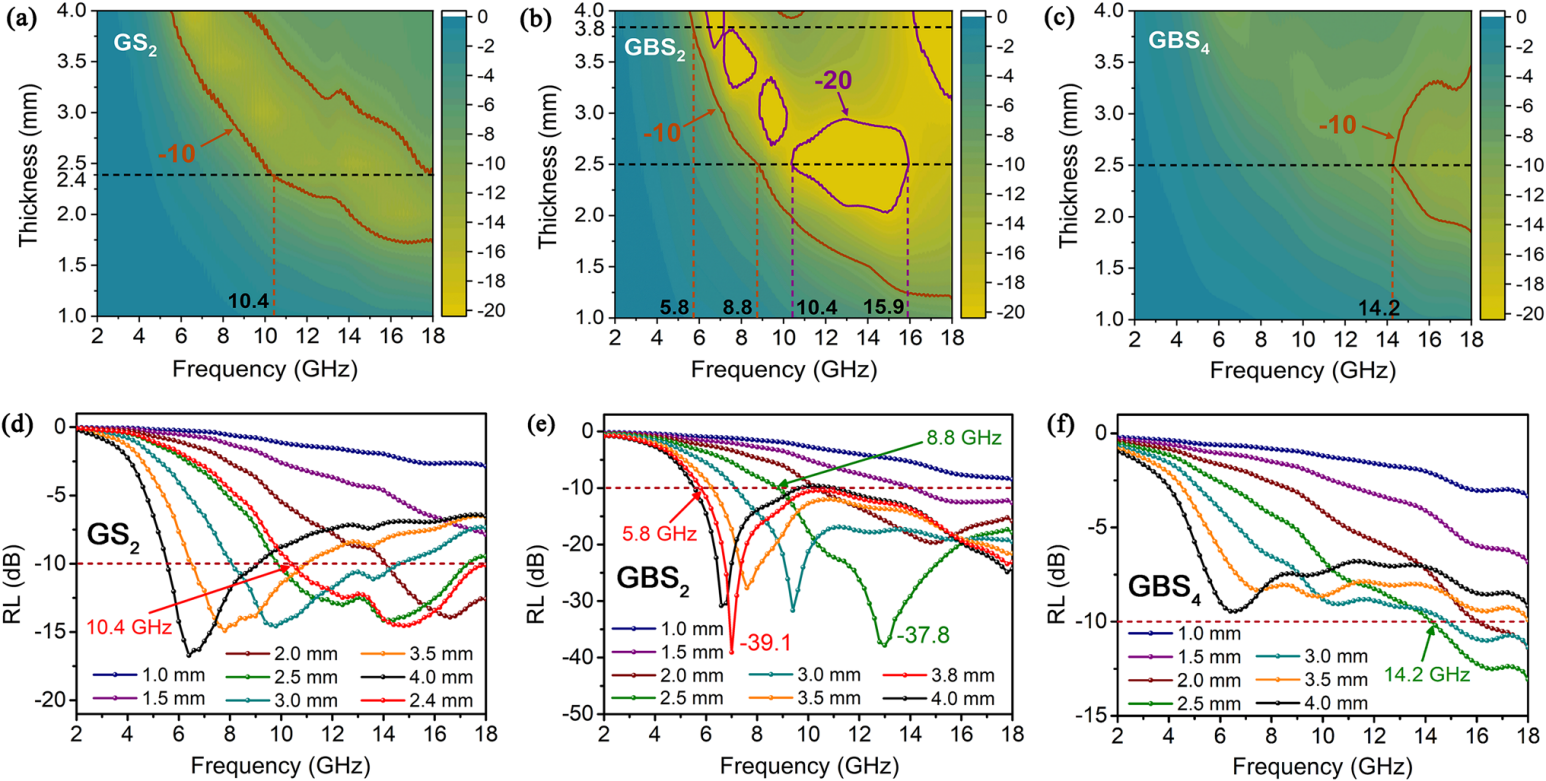
Contour maps of the calculated *RL* values of as-prepared **a** GS_2_, **b** GBS_2_, and **c** GBS_4_ composites with different thicknesses in the frequency range of 2–18 GHz. **d**–**f** Corresponding 2D curves

As shown in Fig. 3a–f, GS_2_ exhibits an EAB of 7.6 GHz at 2.4 mm. GBS_2_ exhibits optimal electromagnetic wave absorption performance with an EAB of 9.2 GHz (from 8.8 to 18.0 GHz) at 2.5 mm. The introduction of hetero-interfacial BN not only reinforces the relaxation process of interfacial polarization and dipole polarization, enhancing the dielectric loss, but also enhances the magnetic loss of the absorber due to the para magnetism of BN. As the increase in SiC_nws_ with a high dielectric constant, the GBS_4_ sample exhibits an EAB of 3.8 GHz at 2.5 mm. This is attributed to the reduction in impedance matching properties, which leads to a reflection of electromagnetic waves at the surface of the composite, resulting in a degradation of the wave absorption performance.

**Fig. 4:**
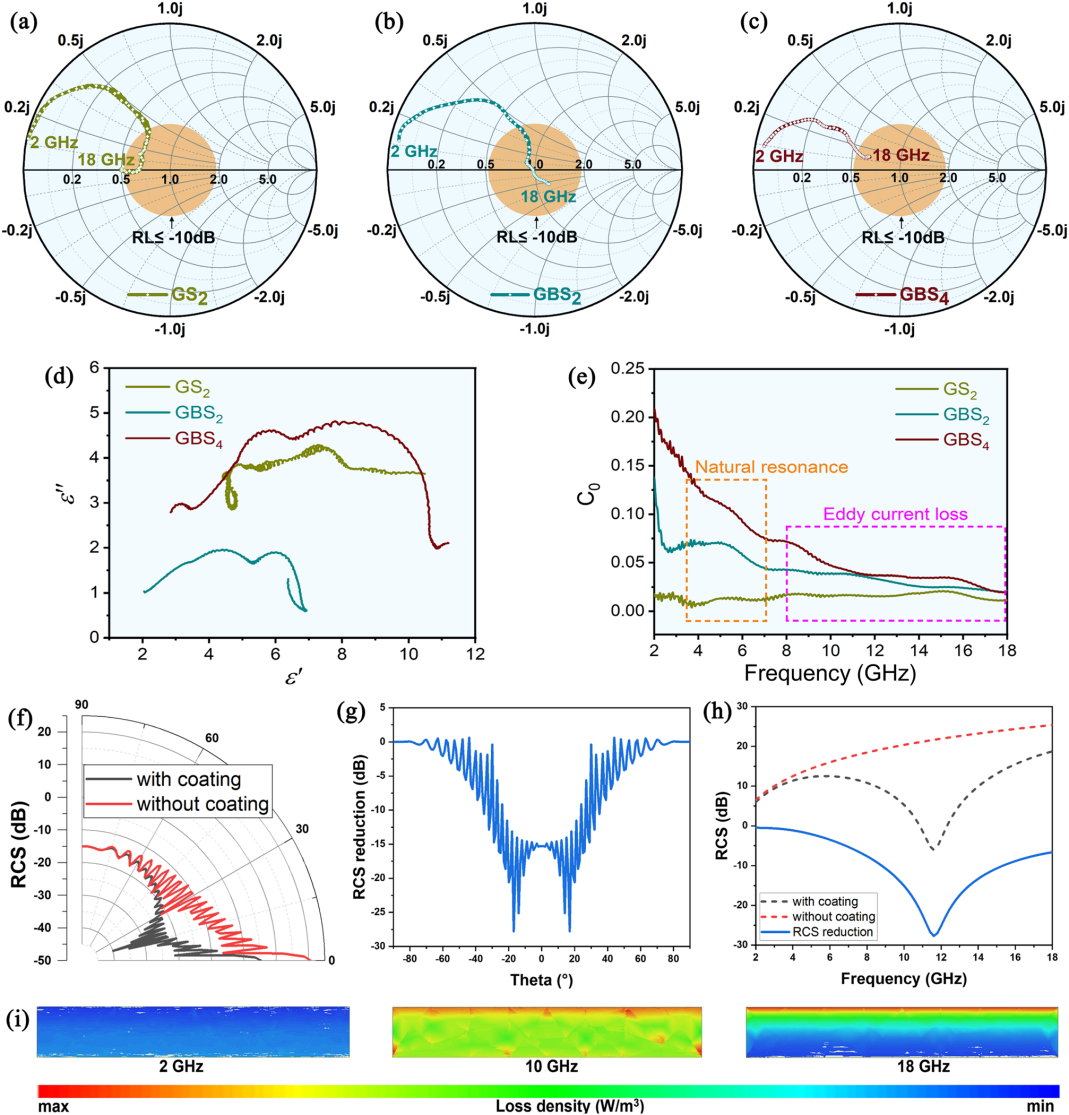
Input impedance of as-prepared **a** GS_2_, **b** GBS_2_, and **c** GBS_4_ composites is shown by the Smith chart (frequency range is 2–18 GHz and thickness is 2.5 mm). **d** Cole–Cole semicircles and **e** C_0_-f curves of as-prepared GS_2_, GBS_2_, and GBS_4_ composites in the frequency range of 2–18 GHz. **f** RCS of the metal plate with and without the applied microwave absorber changes with the incidence angle. **g** RCS reduction changes with the incidence angle. **h** RCS of the metal plate with and without the applied GBS absorbers changes with the frequency. **i** Loss density of electromagnetic waves in applied GBS absorbers at different frequencies

While the absorbers possess strong attenuation capabilities, they also require excellent impedance matching conditions to allow as many electromagnetic waves as possible to enter the interior of the absorbers. As shown in Fig. 4a–c, the optimal electromagnetic wave absorption characteristics can be achieved when the input impedance points fall within the orange circle. The higher dielectric constant and weaker magnetic loss of GS_2_ composites result in input impedance curves that deviate significantly from the orange circle. With the introduction of BN, the permittivity of GBS_2_ material decreases, leading to an enhancement of the impedance matching properties. Consequently, both dielectric loss and magnetic loss are intensified, resulting in the input impedance points falling more inside the orange circle. This leads to a wider absorption bandwidth. As the SiC_nws_ increases, the dielectric constant of the GBS_4_ material rises, resulting in elevated conductive loss. This results in a greater reflection of electromagnetic waves at the surface, which further deviates the input impedance curve from the orange circle. Consequently, the wave absorption performance is diminished.

In order to further analyze the EMWs absorption mechanism of the composites, the Cole–Cole curves and the C_0_-f plots are constructed in Figs. 4d, e and S7. The presence of multiple twisted semicircles in the Cole–Cole curves indicates the existence of multiple relaxation processes [41]. The introduction of BN enriches the interfacial polarization process, and the growth of SiC_nws_ further enriches the network structure, while introducing a large number of heterogeneous interfaces and defects. These interfaces facilitate the movement of free charges under the action of an external electric field, resulting in the formation of localized accumulations and the generation of macroscopic dipole moments. Additionally, impurities and defects existing within graphene, BN, SiC_nws_, as well as the crystal region, amorphous region interface, may also contribute to interfacial polarization. These not only increase the scattering path of electromagnetic waves, but also provide a large amount of polarization loss. Furthermore, the nearly straight line of semicircular distortion is partially attributed to the localized conductive network constituted by SiC_nws_, which generates local currents and notable conductive loss in response to changes in electromagnetic wave frequency. In addition, magnetic loss represents another significant pathway for electromagnetic wave loss. This loss is primarily attributable to natural resonance, exchange resonance, and eddy current loss in the microwave frequency band. As shown in Fig. 4e, the charge distribution in the absorber resonates with the electric field distribution at low electromagnetic field frequencies (2–6 GHz), and the natural resonance is the primary loss mechanism. As the frequency of electromagnetic waves increases, the C_0_ value of each sample gradually stabilizes to a constant, indicating that eddy current loss has become the dominant mechanism [42].

To prove the loss capacity of the as-prepared GBS_2_ absorbers, Fig. 4f shows the variation of radar scattering cross section (RCS) with the incidence angle at 10 GHz of the metal plate with and without the applied microwave absorber. When the electromagnetic wave is normally incident at a 0°, the RCS of the metal plate is the largest, about 20 dBsm. When the as-fabricated microwave absorber is implemented, the RCS is only about 5 dBsm, which is reduced by about 15 dB. Further combined with Fig. 4f, g, it can be seen that with the increase of incidence angle, the reduction effect of the microwave absorber on the RCS of the metal plate first increases and then decreases. When the incidence angle is less than 20°, the RCS reduction in the absorbing coating on the metal plate can reach about 20 dB. When the incidence angle increases further, the attenuation effect of coating on RCS decreases gradually. Figure 4h shows the variation of the RCS of the metal plate with and without the as-prepared GBS_2_ absorber with frequency under the condition of normal incidence. It can be seen that the RCS of the pure metal plate is gradually increasing with the frequency. After the implementation of the GBS_2_ absorber, the RCS of the metal plate decreases rapidly with the frequency in the 8–12 GHz band, and then gradually increases. This shows that under the standard test conditions, the RCS reduction effect of the metal plate in the 8–18 GHz band is significant after the implementation of the microwave absorber, and the reflection reduction is basically consistent with the reflection loss feature of the microwave absorber calculated above.

Furthermore, Fig. 4i studies the loss inside the as-prepared GBS_2_ absorber at different frequencies. At 2 GHz, the loss of electromagnetic waves inside the microwave absorber is generally lower than 1.15 × 10^5^ W m^−3^, and the electromagnetic waves cannot be effectively dissipated, so the reduction in RCS on the metal plate is poor. At 10 GHz, the loss of the microwave absorber to the electromagnetic wave is gradually increased to more than 1 × 10^6^ W m^−3^, and the loss behavior is gradually enhanced. In particular, the surface loss of the microwave absorber is the most significant, close to 1 × 10^7^ W m^−3^. This shows that in addition to the intrinsic loss of the microwave absorber, the resonant effect of 1/4 wavelength plays an important contribution to the electromagnetic wave loss. At 18 GHz, the surface loss of the microwave absorber is gradually enhanced, reaching 2.8 × 10^7^ W m^−3^, while the bottom loss is gradually weakened, which indicates that the resonant absorption at high frequency is the most important form of electromagnetic wave loss of the as-prepared GBS_2_ absorbers.

### Evolution and Mechanism of Microstructure and Microwave Absorption Performance with Different Annealing Time

The thermal stability and the high-temperature wave-absorbing potential of absorbers have received extensive attention from researchers. Herein, the thermal stability of the composites is examined after annealing in a high-temperature air environment. Figure S8 shows the thermogravimetric analysis (TG) image of GA and GBS_2_. As the temperature increased, GA showed three weight losses at about 100, 260, and 486 °C, corresponding to the evaporation of water from GA, the cleavage of oxygen-containing functional groups in graphene oxide, and the oxidation of graphene, respectively. In contrast, the GBS_2_ composite showed only 9.1% weight loss at 787 °C, and the mass retention after annealing at 600 °C for 5 and 10 h was in the range of 89% and 87.8%, respectively. This is attributed to the in situ growth of BN interface, which wraps the GA skeleton, effectively insulating the external air and retarding the GA oxidation process. The mass of the composite gradually increases with a further increase in temperature, which is attributed to the oxidation of the SiC_nws_ to SiO_2_ and the oxidation of BN to B_2_O_3_.

**Fig. 5:**
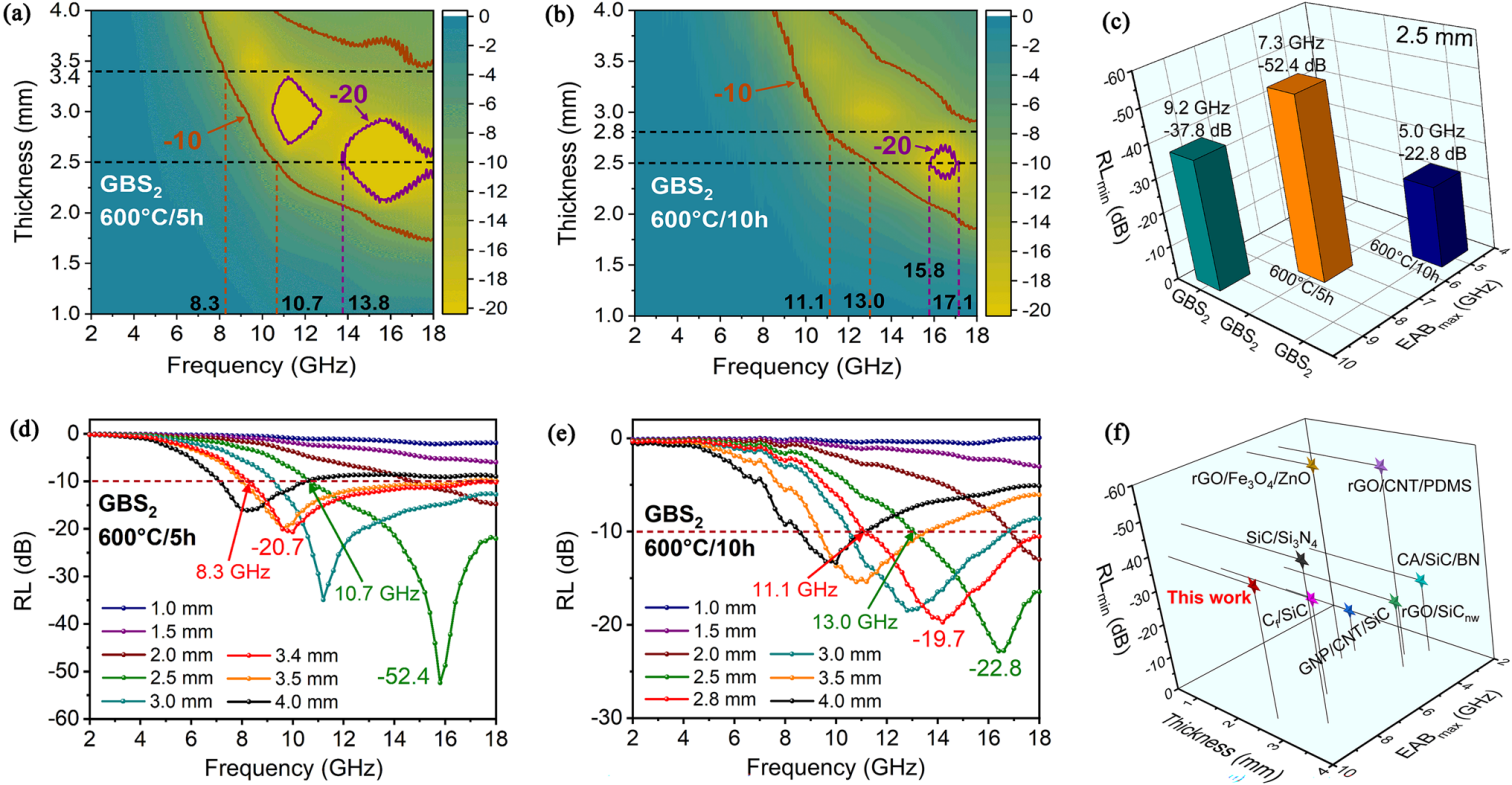
Contour maps of the calculated RL values of as-prepared GBS_2_ composites annealing at 600 °C for **a** 5 h and **b** 10 h in the air with different thicknesses in the frequency range of 2–18 GHz. **c** Comparison of absorption performance of as-prepared GBS_2_ composites annealing at 600 °C for 0, 5, and 10 h **d**, **e** Corresponding 2D curves. **f** Comparison of absorption performance between as-prepared GBS_2_ composites and other typical absorbing materials

The wave absorption performance of the GBS_2_ composite after annealing in air environment at 600 °C is shown in Fig. 5a–e. After annealing for 5 h, the GBS_2_ composite exhibits an EAB of 9.7 GHz (from 8.3 to 18.0 GHz) at 3.4 mm, and the *RL*_min_ decreases to -52.4 dB. The dielectric constant of the composites exhibits minimal variation after annealing, which indicates that that the BN interface effectively impedes the oxidation behavior of graphene. However, both *μ′* and *μ″* of the composites exhibited a decline, which can be attributed to the Curie transition phenomenon of the magnetic components in the composite at high temperatures (Fig. S9). As the annealing time is extended, the EAB decreases to 6.9 GHz (from 11.1 to 18.0 GHz) at 2.8 mm, while the *RL*_min_ becomes -22.8 dB. This is attributed to the partial oxidation of graphene at high temperatures over extended periods of time, which introduces holes and defects that result in slight damage to the microstructure of the composite. Meanwhile, the *C*_0_ of the material decreases with the increase in annealing time. The magnetic particle FeSi undergoes a Curie transition, which results in a dramatic decrease in tan *δ*_*μ*_ of the material, leading to a significant decline in its electromagnetic wave absorption performance. (Figs. S10 and S11).

Moreover, the electromagnetic wave absorption of the GBS_2_ composites, as shown in Fig. 5f and Table S1, is considerably superior to that of the materials that have been previously reported in the literature [20, 24, 31, 43–46]. This indicates that the layered porous structure design and the composites prepared in this work provide a novel approach to the design and preparation of subsequent electromagnetic wave absorption materials.

Besides, the attenuation constant α is also considered to be a key factor affecting the absorbing performance and can be described as follows [47]:

6 $$ \alpha = \frac{\sqrt 2 \pi f}{c}\sqrt {\left( {\mu^{\prime\prime}\varepsilon^{\prime\prime} - \mu^{\prime}\varepsilon^{\prime}} \right) + \sqrt {\left( {\mu^{\prime\prime}\varepsilon^{\prime\prime} - \mu^{\prime}\varepsilon^{\prime}} \right)^{2} + \left( {\mu^{\prime}\varepsilon^{\prime\prime} + \mu^{\prime\prime}\varepsilon^{\prime}} \right)^{2} } } $$

**Fig. 6:**
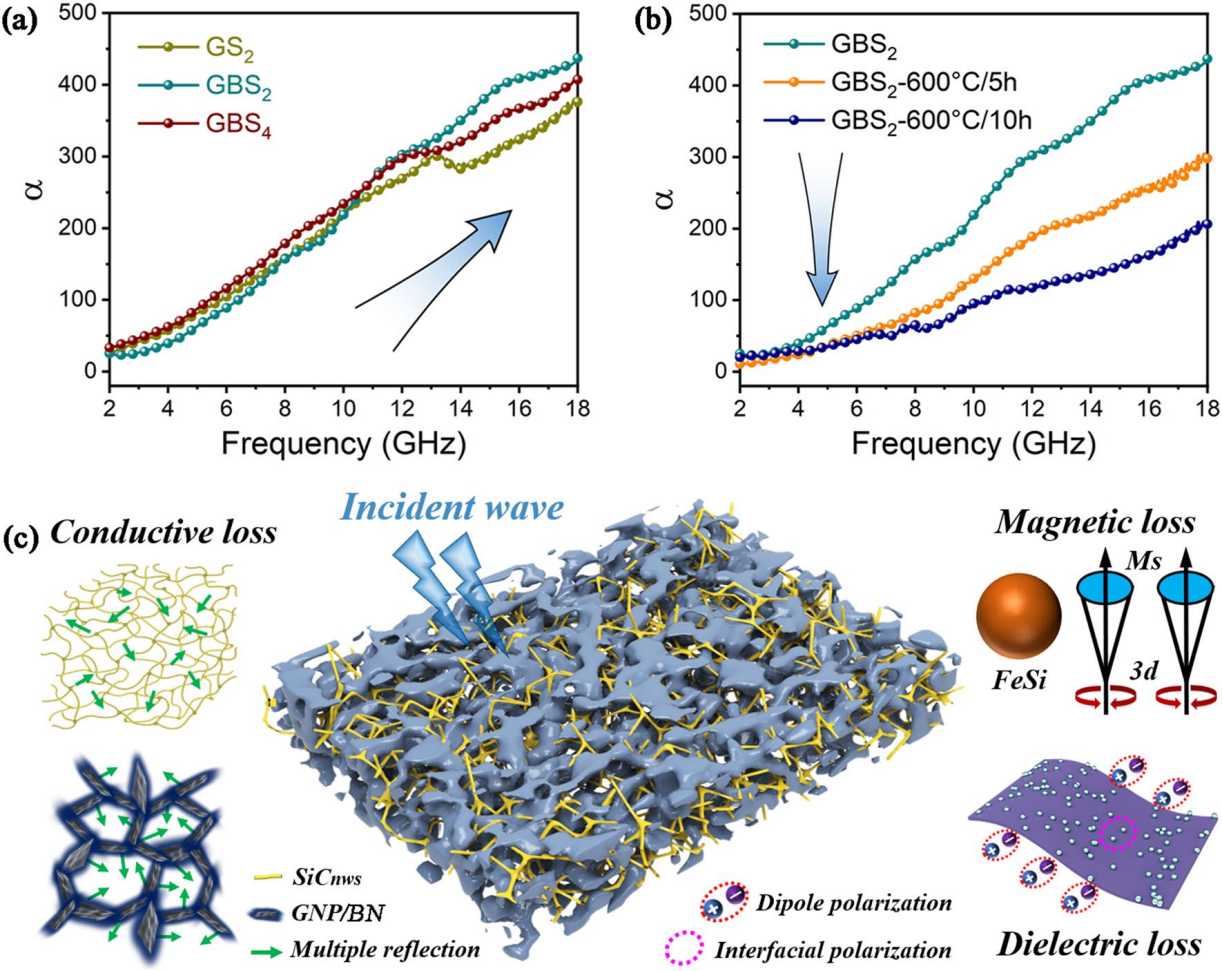
Attenuation constant α of **a** as-prepared GS_2_, GBS_2_, and GBS_4_ composites and **b** as-prepared GBS_2_ composites annealing at 600 °C for 0, 5 and 10 h in the air in the frequency range of 2–18 GHz. **c** Composite microwave absorbing mechanisms of as-prepared GBS_2_ composites

As illustrated in Fig. 6a, b, the attenuation constant α of each sample increases with frequency, demonstrating a positive correlation with the microstructure and electromagnetic properties of the material. As the annealing time increases, α of the GBS_2_ decreases. This is mainly attributed to the significant reduction in the magnetic loss after annealing.

The electromagnetic wave absorption mechanism of GBS_2_ composites can be summarized in several forms, as illustrated in Fig. 6c. Firstly, the design of the porous honeycomb structure not only optimizes impedance matching capability, but also extends the scattering path of electromagnetic waves and enhances the absorption of electromagnetic waves [48]. Secondly, the intrinsic high conductivity of the graphene nanoplates enables the formation of an efficiently connected conductive network. The interconnect SiC_nws_ form a multitude of localized conductive networks. The free movement of electrons is favored to convert electromagnetic waves into Joule heating, which strengthens the conductive loss [49]. Third, the uniform and continuous BN interface and the dispersed FeSi facilitate the construction of a magnetic coupling network, which enhances the space charge polarization and magnetic loss [50]. Fourth, the growth of BN interfaces and SiC_nws_ introduces abundant heterostructure interfaces, while a multitude of vacancies and heteroatoms can become polarization centers and generate dipole polarization, collectively reinforcing the dielectric loss. Based on the combined effect of these mechanisms, GBS_2_ composites exhibit excellent EMW absorption properties and are highly potential wave absorbers.

## Conclusions

In summary, a honeycomb-structured graphene aerogel was constructed by ice template-assisted 3D printing strategy. In situ-grown BN and SiC_nws_ were subsequently incorporated into the aerogel to construct GBS composites with heterogeneous interfaces and a hierarchical porous structure. The multilevel structure and heterogeneous phases of the composites prolong the scattering path of incident EMWs, effectively achieving synergistic optimization of impedance matching and multiple losses. Consequently, the prepared GBS_2_ composite exhibit an EAB_max_ of 9.2 GHz (from 8.8 to 18.0 GHz) at 2.5 mm and a *RL*_min_ of  − 37.8 dB. Furthermore, based on the intrinsic high-temperature resistance of BN and SiC_nws_, the composites exhibit excellent thermal stability. After annealing in air environment at 600 °C for 5 h, the microstructural evolution of the composites resulted in a rising EAB of 9.7 GHz (from 8.3 to 18.0 GHz) at 3.4 mm, indicating a promising potential for high-temperature applications. The microstructure design and preparation in this work offer novel insights into the synergistic optimization of structure and function in low-dimensional materials.

## Supplementary Information

Below is the link to the electronic supplementary material.

Supplementary file1 (DOCX 10238 KB)
