# Supplementary material for: Graphene Aerogel Composites with Self-Organized Nanowires-Packed Honeycomb Structure for Highly Efficient Electromagnetic Wave Absorption
Source: Nanomicro Lett. 2024 Oct 21;17:47. doi: 10.1007/s40820-024-01541-y (PMC11491424; doi:10.1007/s40820-024-01541-y)
Supplement: Supplementary file 1 — Supplementary file1 (DOCX 10238 KB) [file 40820_2024_1541_MOESM1_ESM.docx]

Supporting Information for

**Graphene Aerogel Composites with Self-Oorganized Nanowires-Packed Honeycomb Structure for Highly Efficient Electromagnetic Wave Absorption**

Xiao You^1,3,#,^* , Huiying Ouyang^1,3,5,#^, Ruixiang Deng^2,^*, Qiuqi Zhang^1,3,7^, Zhenzhong Xing^1,3,5^, Xiaowu Chen^1,3^, Qingliang Shan^6^, Jinshan Yang^1,3^, Shaoming Dong^1,3,4,^*

^1^State Key Laboratory of High Performance Ceramics & Superﬁne Microstructure, Shanghai Institute of Ceramics, Chinese Academy of Sciences, Shanghai 200050, P. R. China

^2^Key Laboratory of Inorganic Coating Materials CAS, Shanghai Institute of Ceramics, Chinese Academy of Sciences, Shanghai 200050, P. R. China

^3^Structural Ceramics and Composites Engineering Research Center, Shanghai Institute of Ceramics, Chinese Academy of Sciences, Shanghai 200050, P. R. China

^4^Center of Materials Science and Optoelectronics Engineering, University of Chinese Academy of Sciences, Beijing 100049, P. R. China

^5^School of Physical Science and Technology, ShanghaiTech University, Shanghai 201210, P. R. China

^6^School of Materials Science and Engineering, Zhejiang Sci-Tech University, Hangzhou 310018, P. R. China

^7^University of Chinese Academy of Sciences, Beijing 100039, P. R. China

^#^Xiao You and Huiying Ouyang contributed equally to this work.

Corresponding authors. E-mail: [youxiao@mail.sic.ac.cn](mailto:youxiao@mail.sic.ac.cn) (Xiao You); [dengruixiang@mail.sic.ac.cn](mailto:dengruixiang@mail.sic.ac.cn) (Ruixiang Deng); [smdong@mail.sic.ac.cn](mailto:smdong@mail.sic.ac.cn) (Shaoming Dong)

**Supplementary Figures and Table**


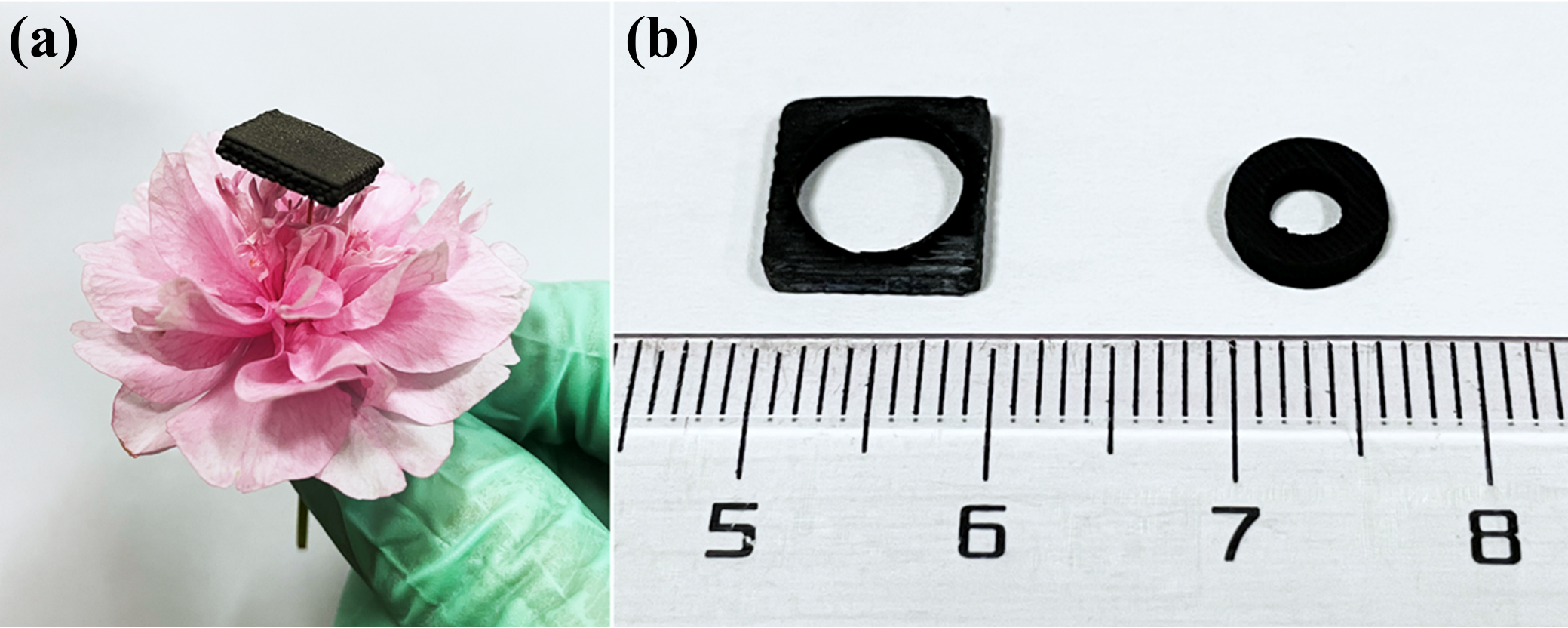


**Fig. S1** The optical images of as-prepared **a** lightweight GA and **b** GBS composites. The circular structure was manufactured by laser cutting with an inner diameter of 3 mm and an external diameter of 7 mm


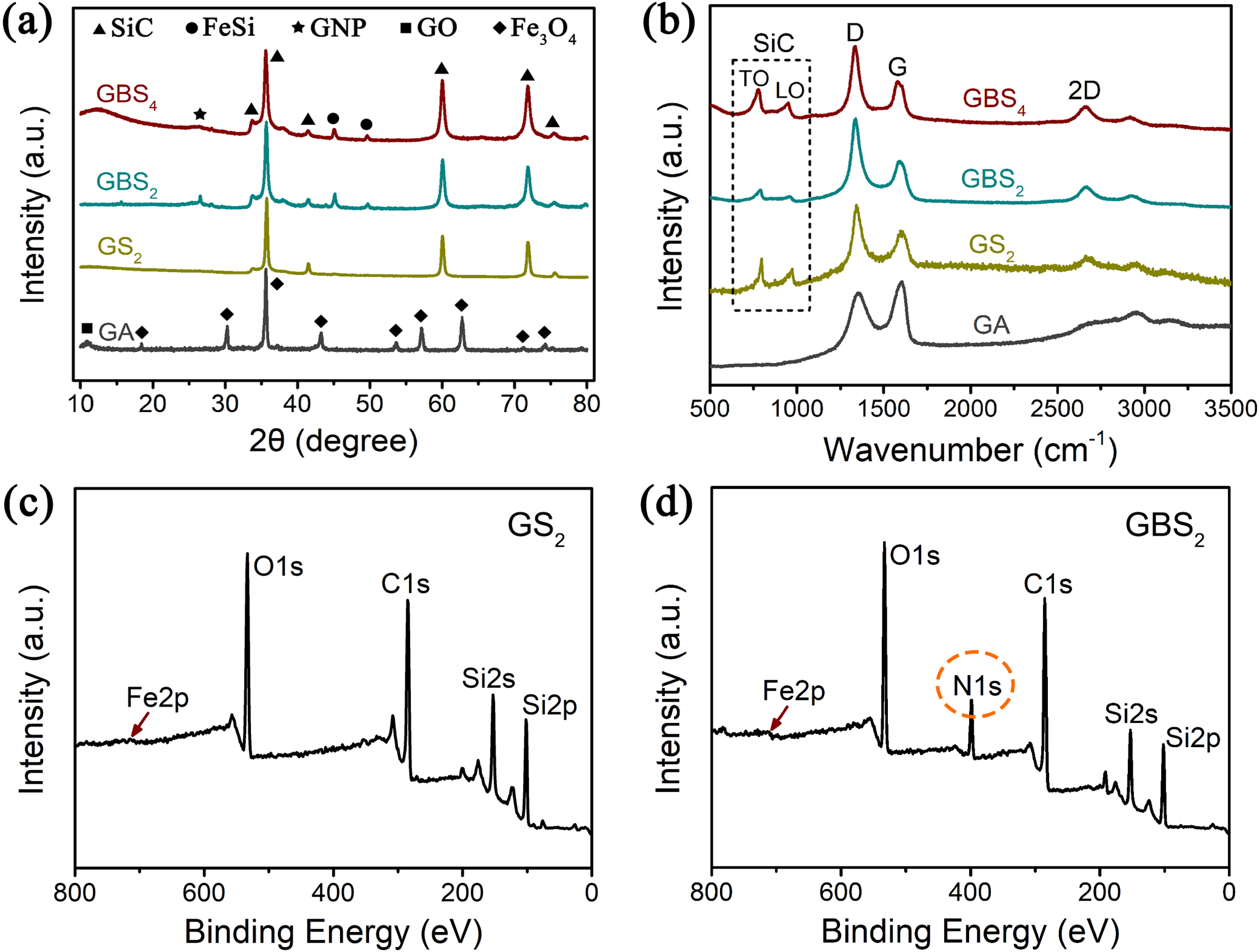


**Fig. S2** Characterizations chemical composition: **a** XRD pattern. **b** Raman spectra. **c, d** Survey spectrum of XPS spectra of as-prepared GS_2_ and GBS_2_ composites


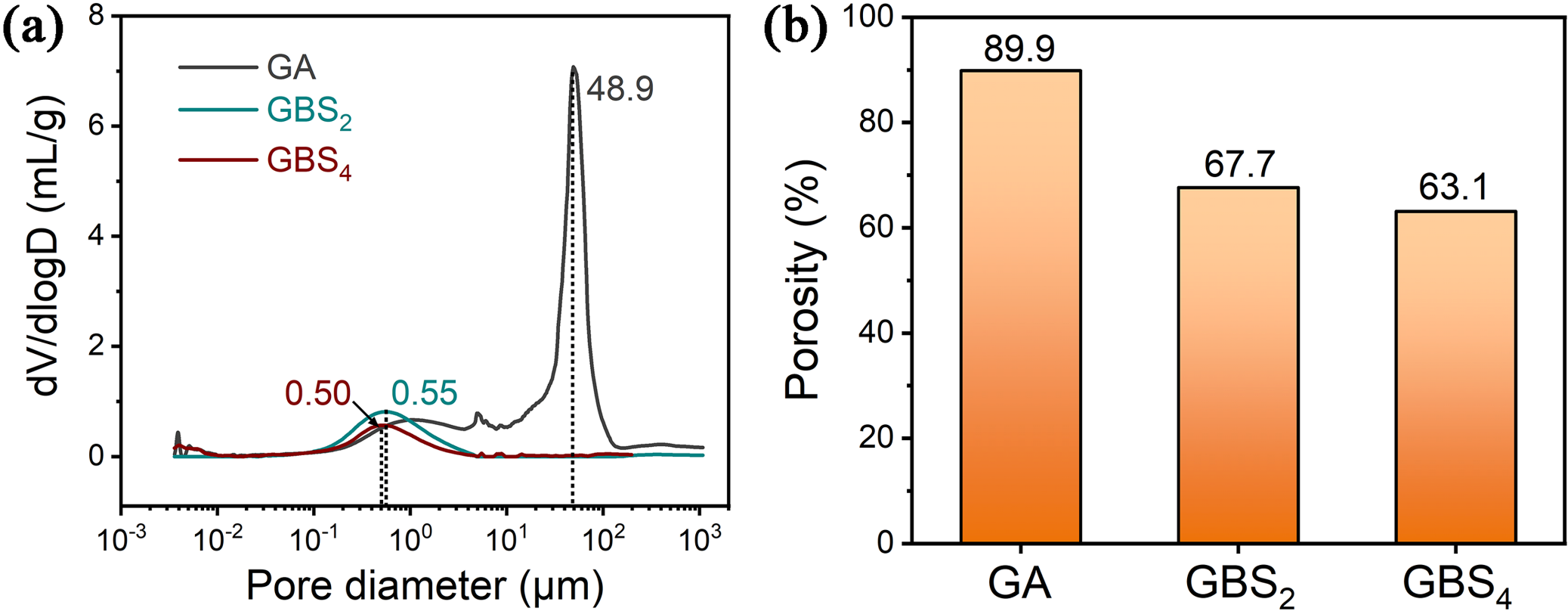


**Fig. S3 a** Pore size distribution and **b** porosity of as-prepared GA, GBS_2_, and GBS_4_ composites


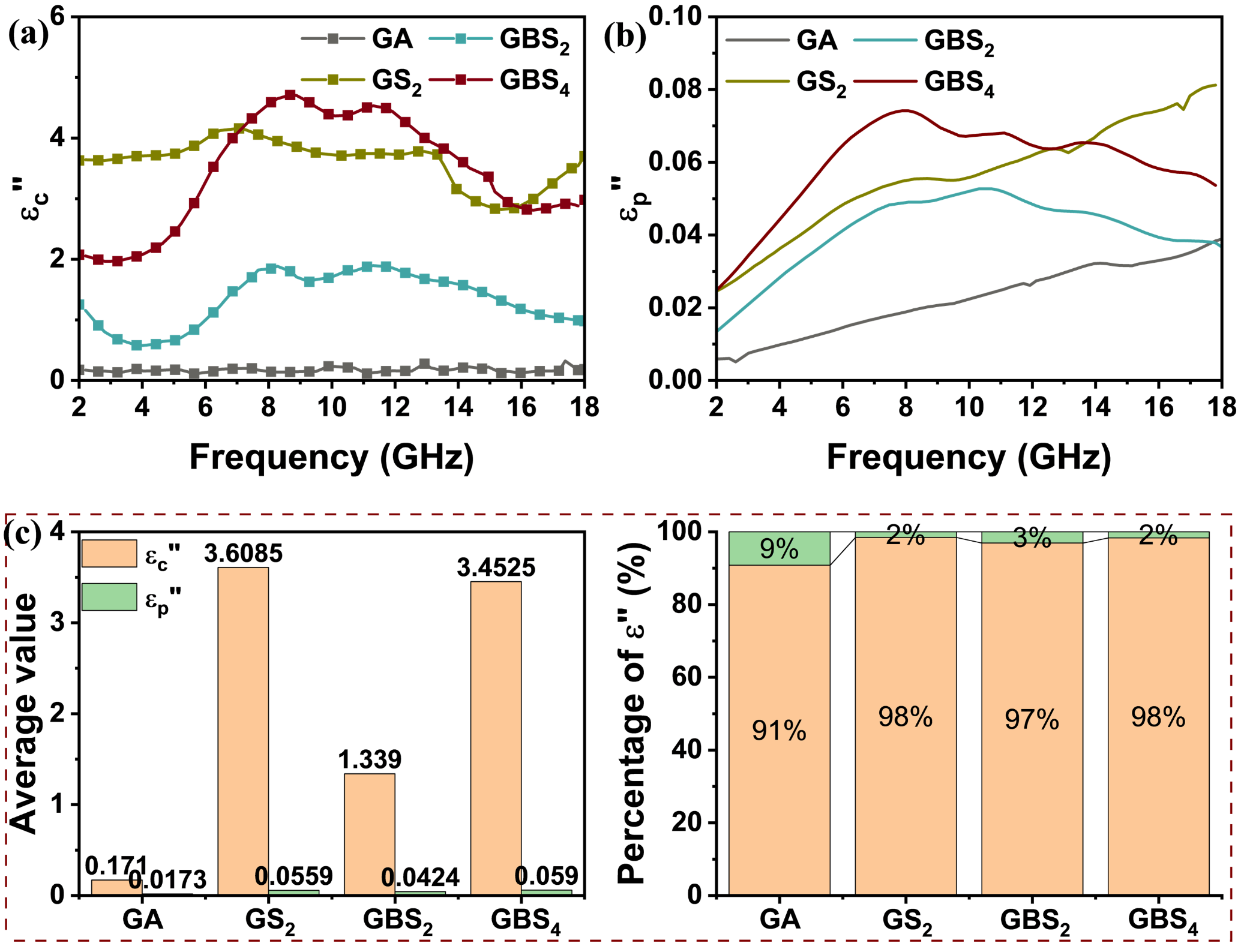


**Fig. S4** Frequency dependence of **a** *ε_c_''*, **b** *ε_p_''*, **c** average value of *ε_c_''*, *ε_p_''* and percentage of *ε''*


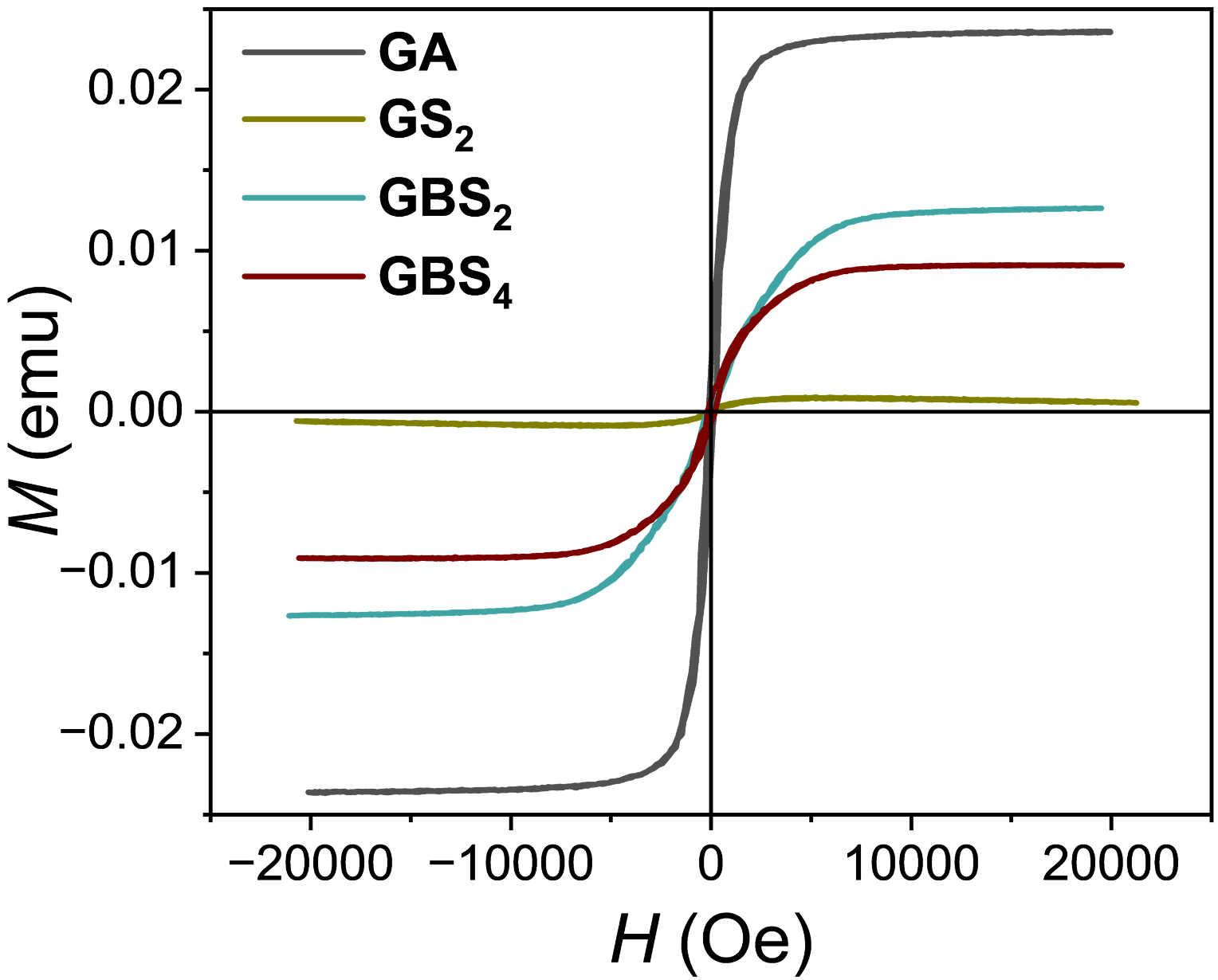


**Fig. S5** Magnetic hysteresis loop of as-prepared GA, GS_2_, GBS_2_, GBS_4_ composites


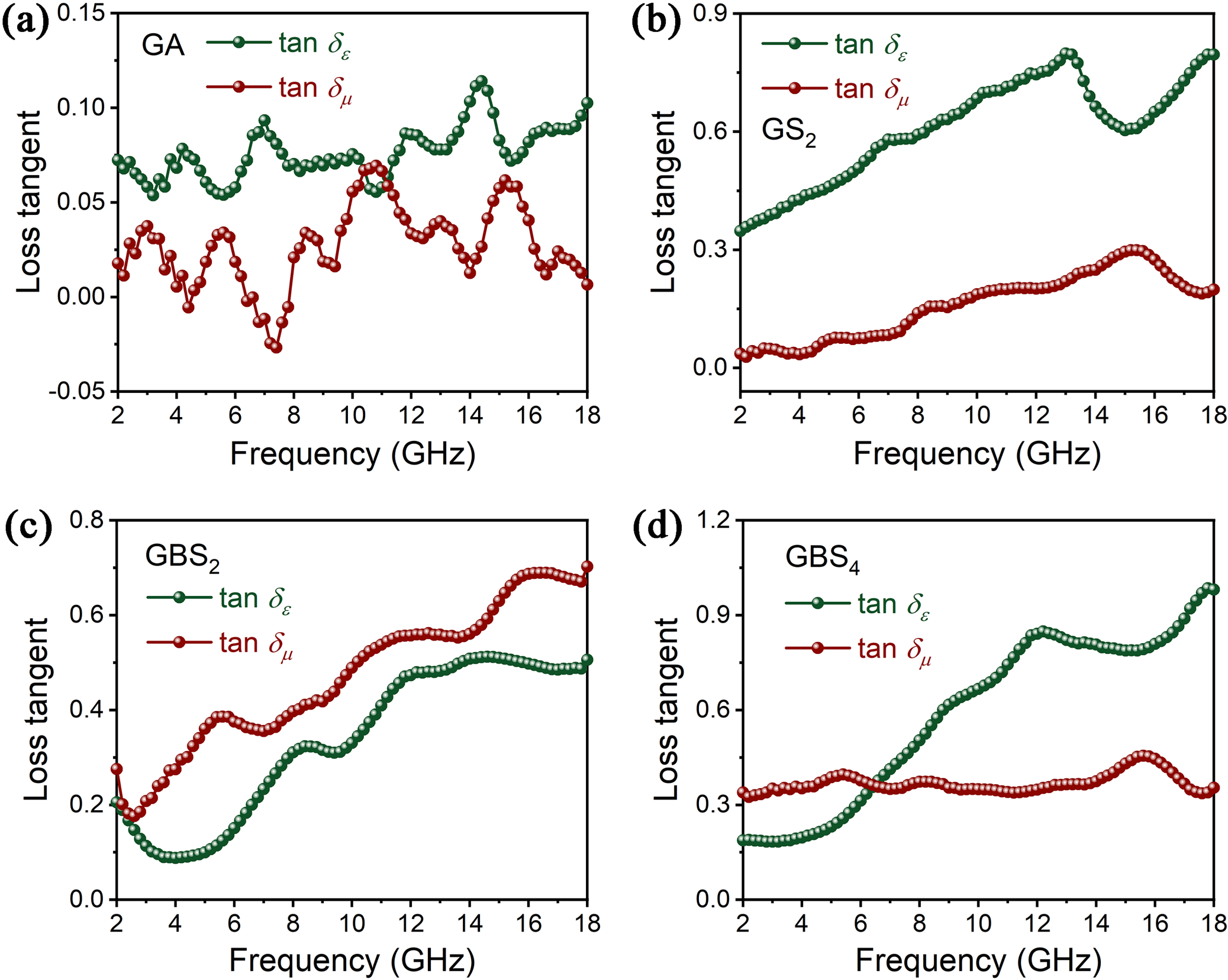


**Fig. S6** Dielectric and magnetic tangent loss as a function of frequency of as-prepared **a** GA, **b** GS2, **c** GBS_2_, and **d** GBS_4_ composites


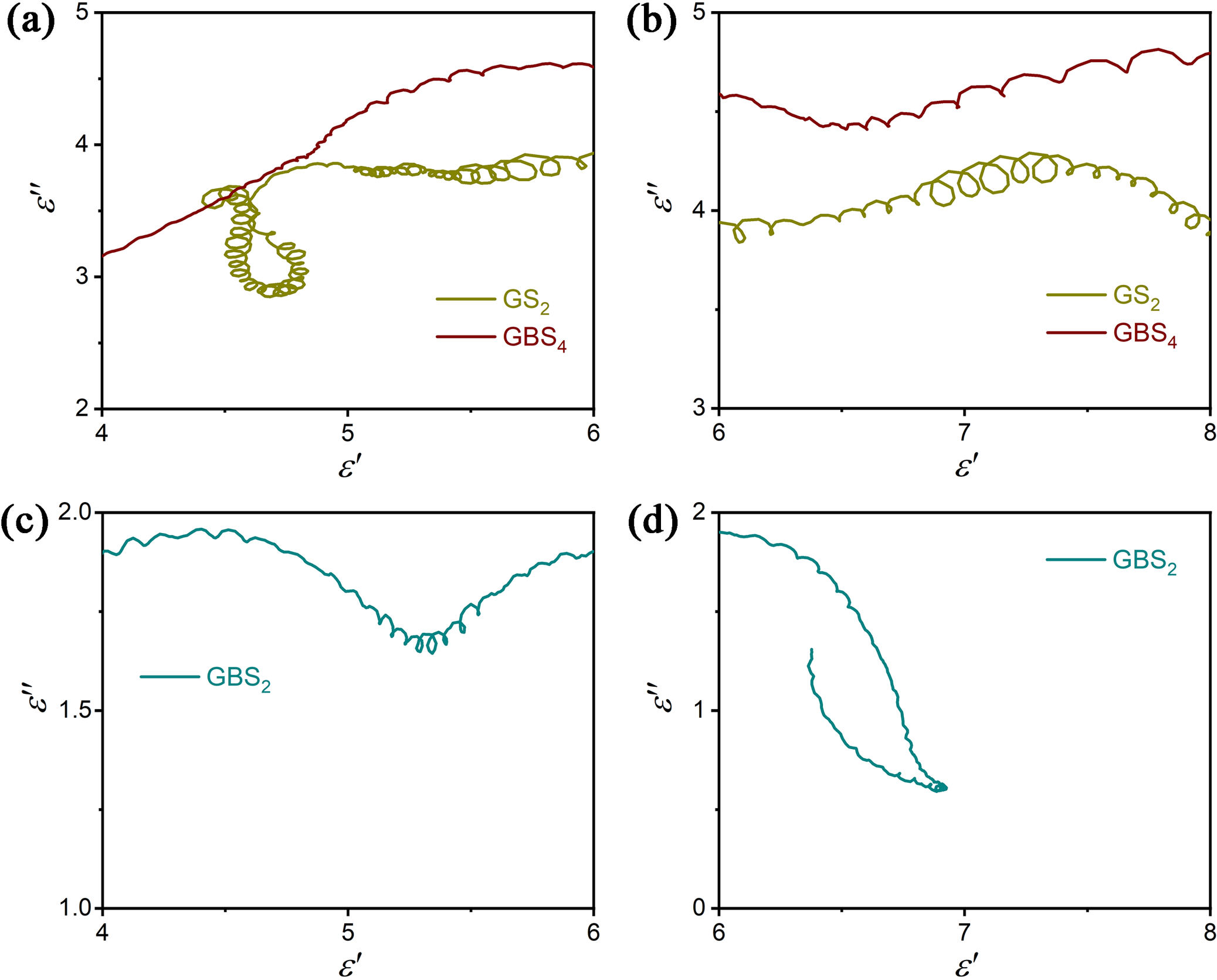


**Fig. S7** The enlarged Cole-Cole semicircles of as-prepared GS_2_, GBS_2_, and GBS_4_ composites with different frequency range


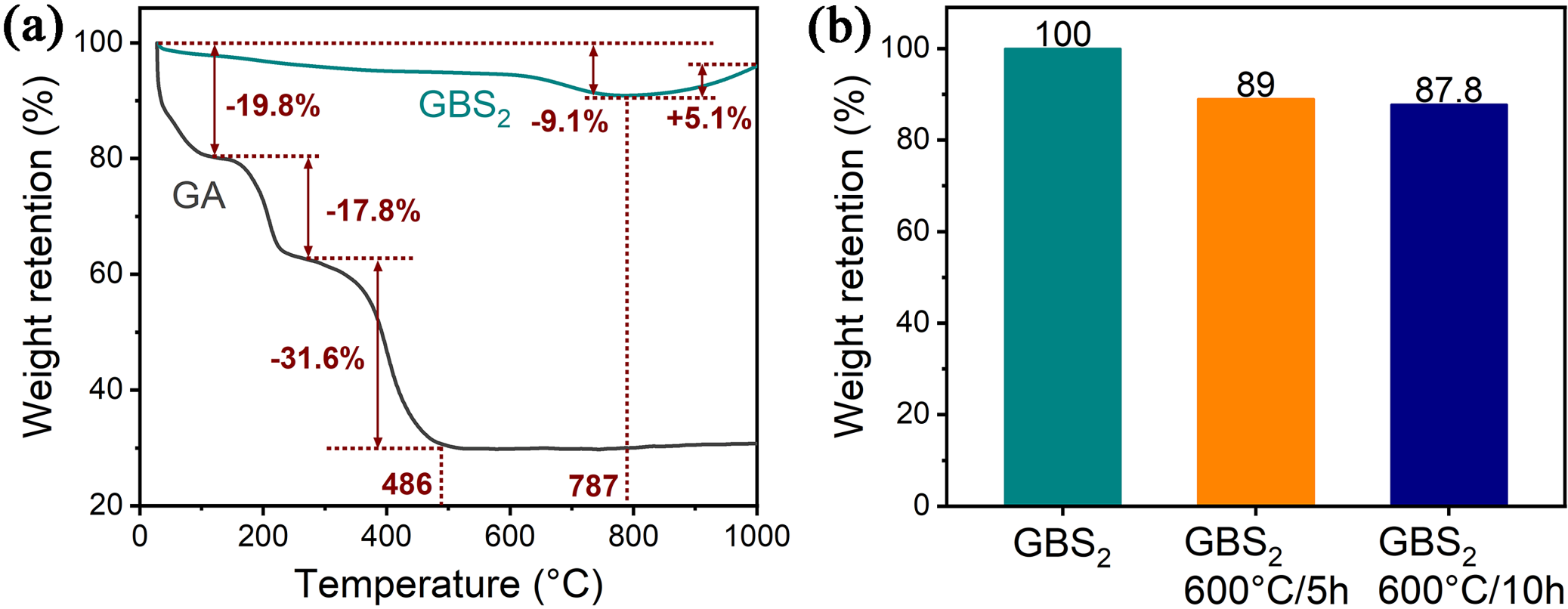


**Fig. S8 a** TGA curves of as-prepared GA and GBS_2_ composites from RT to 1000 ℃. **b** Weight retention of as-prepared GBS_2_ composites annealing at 600 ℃ for 0 h, 5 h, and 10 h


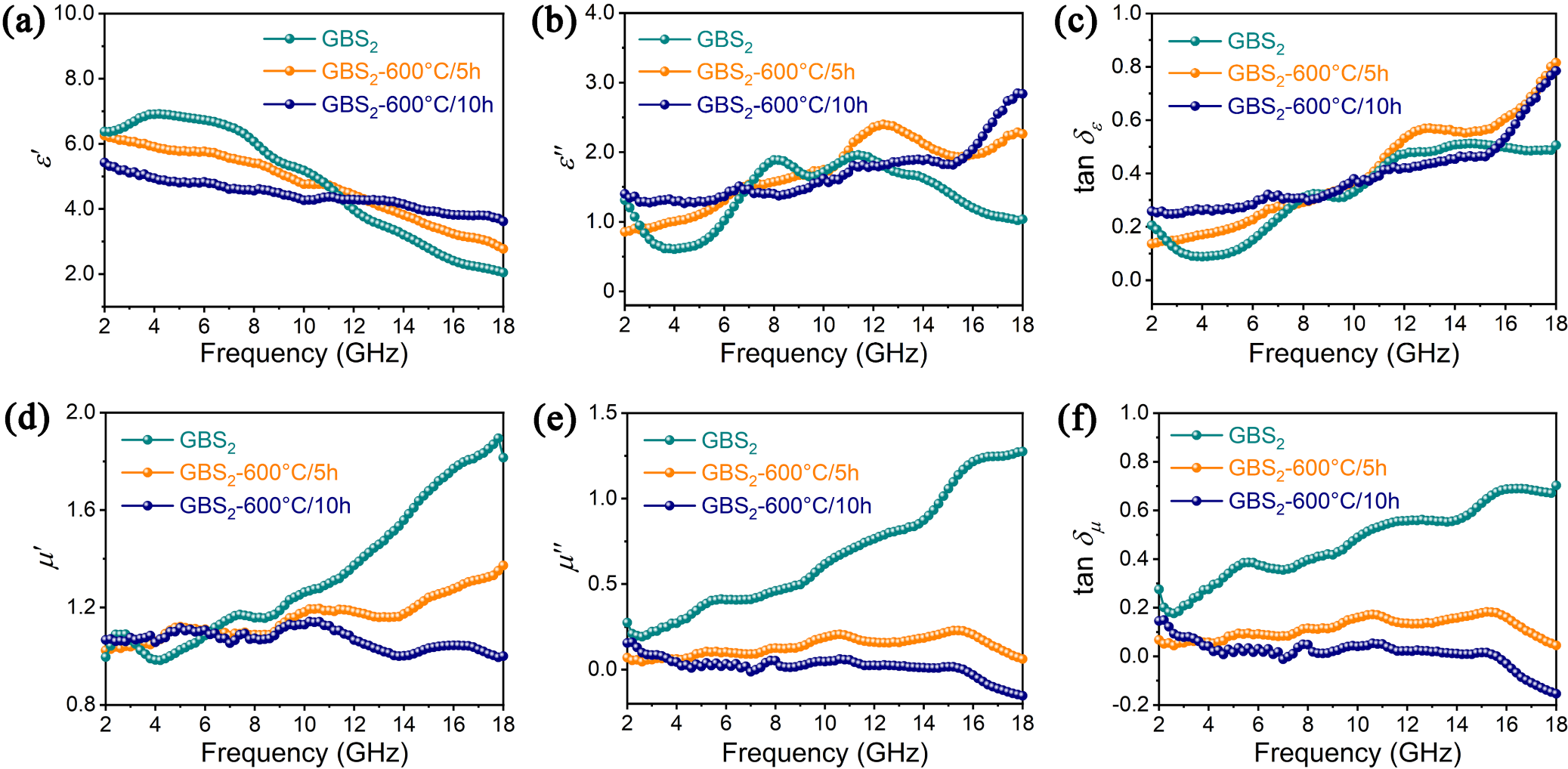


**Fig. S9 a** The real part *ε*′, **b** imaginary part *ε*″ and **c** tangent loss tan *δ_ε_* as a function of frequency of as-prepared GBS_2_ composites annealing at 600 ℃ for 0 h, 5 h, and 10 h. **d** The real part *μ*′, **e** imaginary part *μ*″ and **f** tangent loss tan *δ_μ_* as a function of frequency of as-prepared GBS_2_ composites annealing at 600 ℃ for 0 h, 5 h, and 10 h


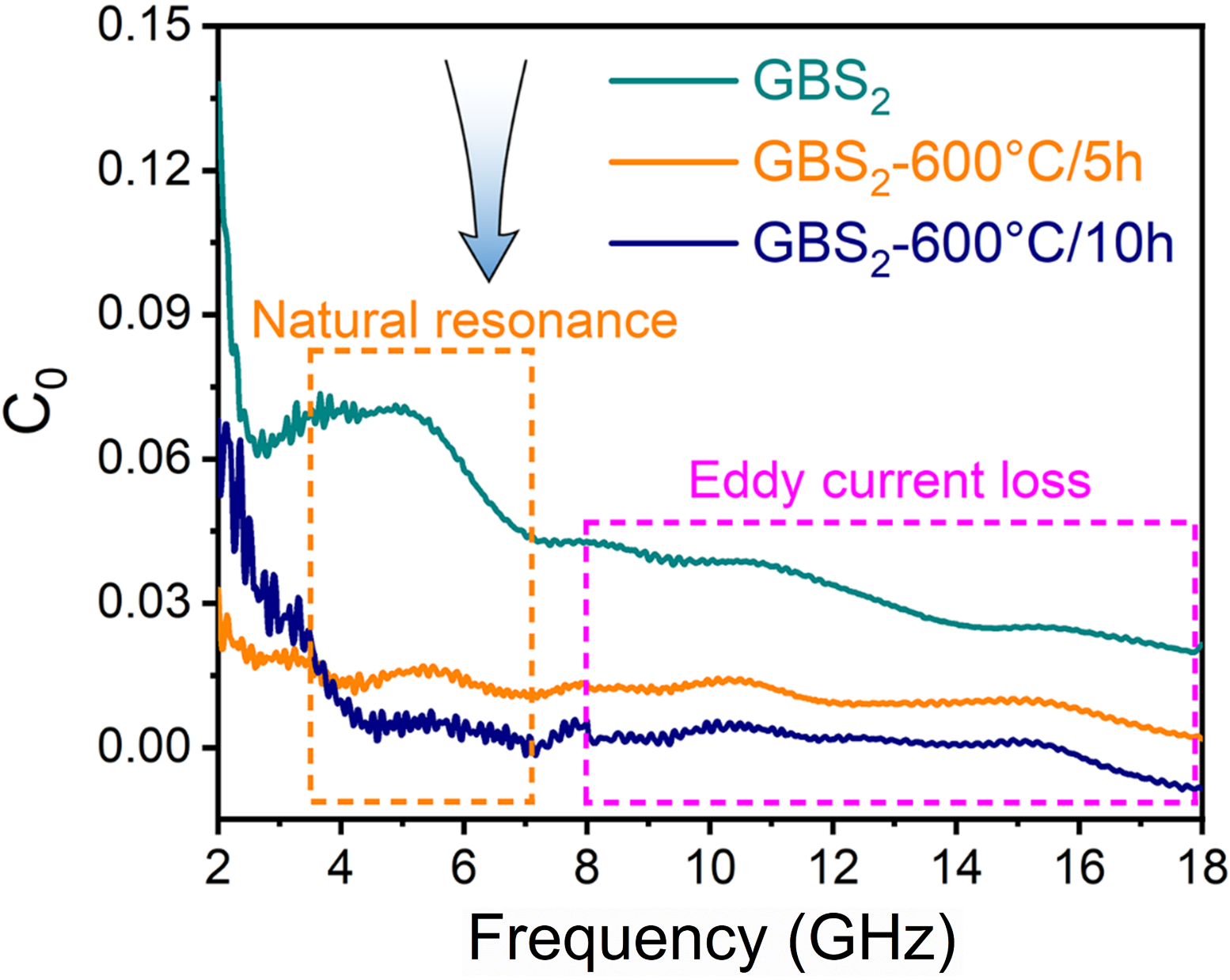


**Fig. S10** C_0_-f curves of as-prepared GBS_2_ composites annealing at 600 ℃ for 0, 5, and 10 h


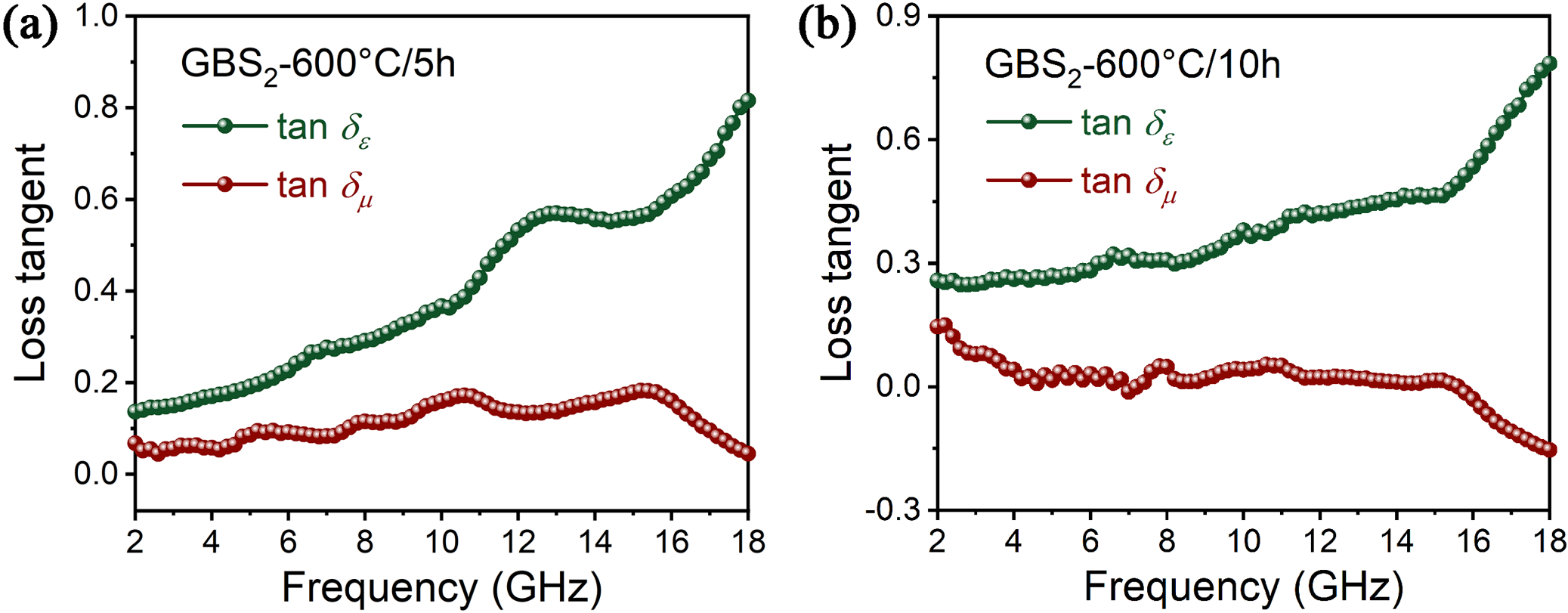


**Fig. S11** Dielectric and magnetic tangent loss as a function of frequency of as-prepared GBS_2_ composites annealing at 600 ℃ for **a** 5 h and **b** 10 h

**Table S1** Absorption performance comparison of this work with other EMW absorbers [20, 24, 31, 43–46]

| Samples | Thickness (mm) | RL_min_ (dB) | EAB (GHz) | References |
| --- | --- | --- | --- | --- |
| CA/SiC/BN | 3.0 | -21.5 | 2.8 | [20] |
| GNP/CNT/SiC | 2.0 | -48.7 | 4.3 | [24] |
| rGO/SiC_nw_ | 3.0 | -19.6 | 4.2 | [31] |
| rGO/Fe_3_O_4_/ZnO | 2.0 | -57.0 | 5.0 | [43] |
| SiC/Si_3_N_4_ | 3.2 | -45.0 | 8.4 | [44] |
| rGO/CNT/PDMS | 2.8 | -55.0 | 3.5 | [45] |
| C_f_/SiC | 2.2 | -53.7 | 7.1 | [46] |
| GBS_2_ | 2.5 | -37.8 | 9.2 | This work |

(The references are numbered in accordance with the sequence in the main manuscript)
